# Multilevel Factors Influencing Linkage to HIV Care in a High HIV Prevalence District in South Africa: A Mixed Methods Approach

**DOI:** 10.1007/s10461-026-05140-3

**Published:** 2026-04-21

**Authors:** Edward Nicol, Wisdom Basera, Thubelihle Mathole, Eunice Turawa, Mbuzeleni Hlongwa, Carl Lombard, Debbie Bradshaw

**Affiliations:** 1 Burden of Disease Research Unit, South African Medical Research Council, Cape Town, South Africa; 2 Division of Health Systems and Public Health, Stellenbosch University, Cape Town, South Africa; 3 School of Public Health and Family Medicine, University of Cape Town, Cape Town, South Africa; 4 School of Public Health, University of Western Cape, Cape Town, South Africa; 5 Public Health, Societies and Belonging, Human Sciences Research Council, Pretoria, South Africa; 6 School of Nursing and Public Health, University of KwaZulu-Natal, Durban, South Africa; 7 Biostatistics, South African Medical Research Council, Cape Town, South Africa

**Keywords:** HIV care continuum, Linkage to care, HIV epidemiology, Universal test and treat, South Africa

## Abstract

Timely linkage to HIV care is essential for achieving South Africa’s treatment goals, yet significant gaps persist in the early stages of the HIV care continuum. This study examined multilevel factors influencing linkage to care in a high HIV-prevalence rural district. A prospective convergent mixed-methods cohort study was conducted over 21 months among 1,194 adults newly diagnosed with HIV across 18 facilities in KwaZulu-Natal. Quantitative analyses (Pearson’s chi-square, Mann-Whitney tests, and logistic regression) were used to identify factors associated with linkage to care within three months of diagnosis. Qualitative data from in-depth interviews with 38 purposively selected participants were analysed thematically and triangulated with quantitative findings to provide contextual insights. Overall, 83% of participants linked to care within three months. Linkage was significantly higher among those diagnosed at hospitals (OR = 4.90; 95% CI: 1.87–12.82; *p* = 0.003), while diagnosis at mobile clinics showed borderline significance. Among women, later sexual debut (≥ 17 years) was associated with increased likelihood of timely linkage. In contrast, condom use at last sex (OR = 0.50; 95% CI: 0.32–0.79; *p* = 0.03) and substance use (OR = 0.67; 95% CI: 0.47–0.96; *p* = 0.05) were associated with lower linkage. Qualitative findings highlighted supportive family environments and positive provider interactions as key facilitators of linkage, while stigma, fear of disclosure, confidentiality concerns, preference for traditional or spiritual healing, and health system challenges, including lost clinic files and poor counselling, emerged as significant barriers. These findings underscore the need for patient-centred, stigma-free, and responsive health systems to improve timely linkage to HIV care in high-burden settings.

**Supplementary Information:**

The online version contains supplementary material available at https://doi.org/10.1007/s10461-026-05140-3.

## Introduction

An estimated 7.8 million South Africans were living with HIV (PLHIV) in 2024 [1], with approximately 170,000 new infections reported that year [2]. Among PLHIV, 95% were aware of their status, and 85% of those diagnosed were on antiretroviral therapy (ART). Among individuals on ART, 92% had achieved viral suppression, defined as a latest viral load of less than 400 copies/cm³ [2]. To accelerate progress towards universal HIV treatment coverage and ending AIDS as a public health threat, the World Health Organization (WHO) endorsed the Universal Test-and-Treat (UTT) strategy in 2015, recommending immediate ART initiation for all HIV-positive individuals regardless of CD4 count or clinical staging [3–5]. South Africa adopted a phased rollout of the UTT strategy from September 2016 [6] to reduce the high rate of individuals newly diagnosed with HIV who were being lost to follow-up before ART initiation. Despite substantial investments in the expansion of HIV testing services and differentiated models of care, gaps persist in the early stages of the HIV care continuum.

Linkage to care, remains critical for ART initiation and sustained engagement in care [7]. Under UTT, individuals who test HIV positive receive same-day ART counselling, laboratory assessments, and initiation of treatment when clinically and psychologically ready, with delays occurring only in cases of acute illness or when individuals choose to postpone treatment [8, 9]. Early linkage to HIV care, particularly same-day initiation, minimizes the risk of early attrition and enhances both individual-, population-, and health system-level health outcomes [10].

Studies have consistently identified multiple structural, psychosocial and health systems barriers to timely linkage, including long clinic wait times, limited service availability, clinic workload, low health literacy, transportation costs, and HIV-related stigma [10–14]. A systematic review by Govindasamy et al. (2012) highlighted transport cost and long distance to health facilities as the most frequently cited barriers [15], findings reinforced by Sanga et al. (2018), who found that increasing distance significantly reduced the likelihood of linkage [14]. Other barriers include fear of disclosure, staff shortages, previous negative experiences with the health system, and concerns about ART side effects [15, 16].

Despite the well-documented importance of rapid linkage to care for improved treatment outcomes [17–19], individual, social, and health system barriers continue to undermine efforts to initiate ART promptly following diagnosis across many settings. Against this backdrop, the present study aimed to explore multilevel factors influencing linkage to HIV care among newly diagnosed adults in a high-prevalence rural district of South Africa. Building on earlier work focused on enhancing linkage to care [16, 20], this paper uses a mixed methods approach to capture both quantitative patterns and qualitative experiences that shape engagement in the early stages of the HIV care continuum.

## Methods

### Study Design

**Fig. 1:**
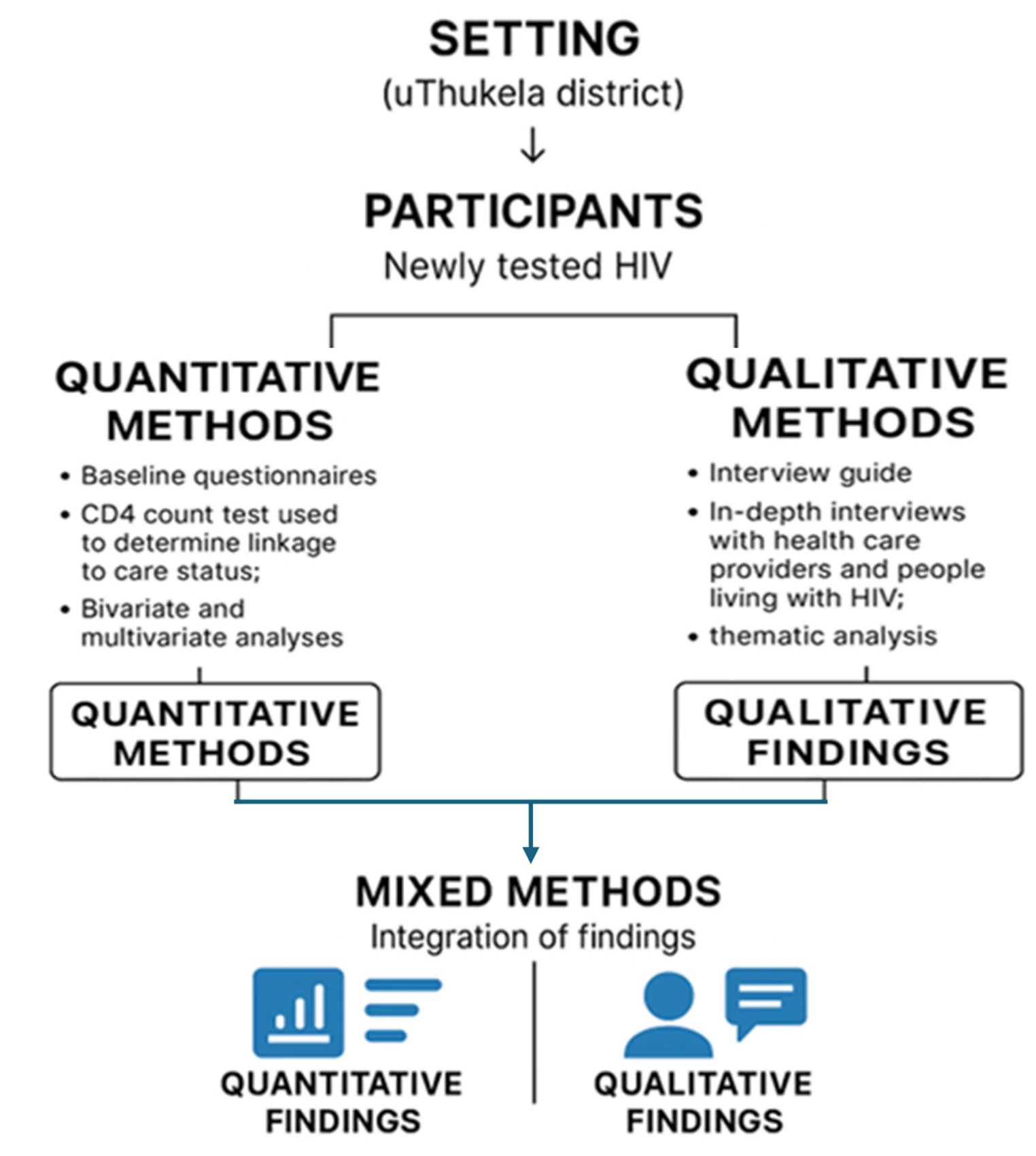
Convergent mixed methods design

We utilized a convergent mixed methods design [21, 22] for this 21-month observational prospective cohort study conducted in KwaZulu-Natal, South Africa. As illustrated in Fig. 1, the study combined quantitative and qualitative components that were implemented concurrently and later integrated during analysis. Mixed methods were used to gain a better understanding of the research problem [23, 24]. The rationale for combining both types of data within one study is that neither quantitative nor qualitative methods are sufficient, by themselves, to capture the trends and details of the complex multilevel factors influencing linkage to HIV care in our study settings. When used in combination, quantitative and qualitative methods complement each other and enable more robust analysis by leveraging the strengths of each [23, 25].

### Study Setting

The study was conducted in the uThukela District Municipality (DM) in KwaZulu-Natal, South Africa, a predominantly rural district with an estimated HIV prevalence of 25.4% (23.4–27.6%) as of 2024, higher than the national prevalence of 17.2% [26]. The district comprises three local municipalities: Alfred Duma (51% of the population), which includes Ladysmith, the administrative seat of both the local and district municipalities; Inkosi Langalibalele (30%), with mixed rural and urban populations; and Okhahlamba (19%), characterized by mountainous terrain, poor road infrastructure, and limited access to fixed clinics [27].

During the study period, 56 public-sector facilities provided HIV services, including antiretroviral therapy (ART), excluding correctional and non-medical sites. Eighteen facilities were selected based on mean monthly HIV-positive test yield, including three gateway clinics, ten fixed primary health care clinics, three mobile units, one community health centre, and a hospital outpatient department. A detailed description of the study setting has been extensively reported in complementary publications [16, 20].

### Sampling Approach and Enrolment Eligibility Criteria

Both quantitative and qualitative sampling approaches were used. For the quantitative phase, a sample size of 1,080 participants was determined to estimate the proportion of individuals diagnosed with HIV who linked to care. In this study, linkage to HIV care was defined as completion of the first clinical visit within three months of an HIV-positive diagnosis, as verified through a record in TIER.Net, a system of three Interlinked electronic registers used nationally in South Africa to monitor and manage patients receiving HIV and tuberculosis (TB) care [28].

The study hypothesized, based on a previous surveillance data showing an average linkage to care of 62% [29] that UTT could increase linkage to care to 72% (a 10% improvement). Assuming cluster randomization with an intraclass correlation coefficient of 0.02, 80% statistical power, and a 0.05 significance level, a minimum sample size of 18 clusters with 50 participants per cluster (900 participants in total) was required to detect a difference in linkage to care proportions (Table 1). Accordingly, 18 high-performing HIV testing facilities were purposely selected from the District Health Information System (DHIS), each reporting more than 4,000 HIV tests per month and an average of at least 10 newly identified HIV-positive cases monthly. Approximately 10 participants per facility per month were expected to be recruited over six months, yielding about 60 participants per facility and a potential total of 1,080 participants, exceeding the required sample size. Convenience sampling was used to enrol HIV-positive participants until the target sample size were met. Detailed sample size calculations have been reported elsewhere [20, 30].

**Table 1:** Power and sample size calculations using the coefficient of variation between clusters in the linkage to care study

Matched pairs										
p_1_	p_2_	delta	alpha	Beta	k_1_	k_2_	m_1_	m_2_	rho	N
0.62	0.72	0.10	0.05	0.80	9	9	50	50	0.02	900

Where p - linkage to care proportions; delta – difference between proportions; alpha - z values used for calculating type 1 error; beta - z value used for calculating power; rho – inter-cluster correlation; m – average cluster size and k - number of clusters. N - sample size for study period allowing for cluster randomization

**Fig. 2:**
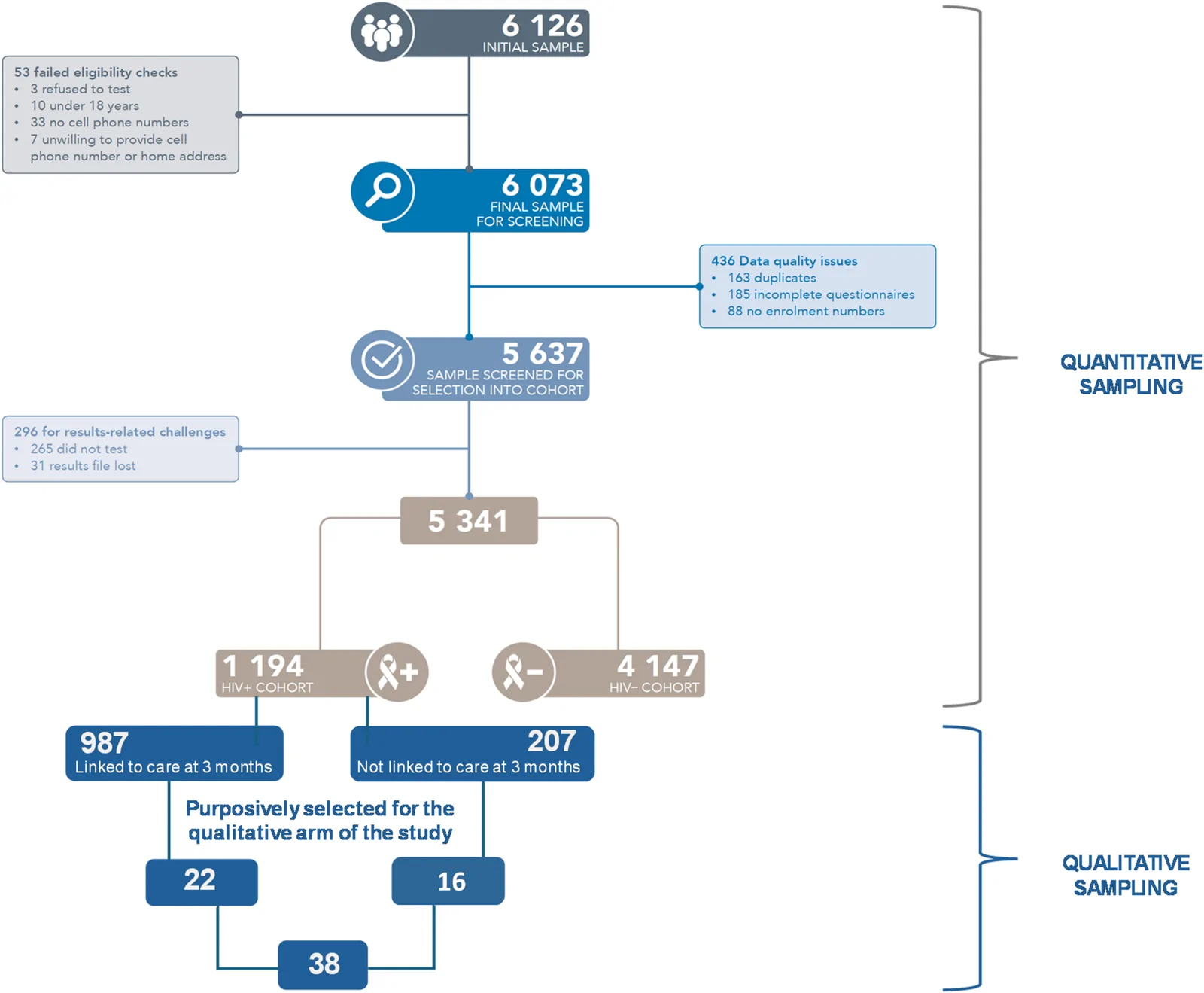
Consort diagram detailing the sampling approach for the recruitment of participants into the linkage to care study in uThukela district

The qualitative component used a cross-sectional exploratory design, comprising in-depth interviews with a purposively selected subsample of 38 participants drawn from the cohort of 1,194 HIV-positive patients recruited in the quantitative study. Selection was based on the outcome of the 4-month follow-up survey: 22 participants who successfully linked to care within three months and 16 who did not (Fig. 2). Maximum variation sampling was employed to ensure diverse representation across linkage status, age, gender, marital status, employment, geographic area, and health facility location. A detailed description of these processes has been published elsewhere [16].

### Inclusion and Exclusion Criteria

Participants were eligible for enrolment if they were 18 years or older, willing to provide informed consent, planned to undergo HIV test at one of the 18 participating HIV testing and treatment facilities, and own a cell phone with a willingness to share contact details. Exclusion criteria included being under 18 at enrolment, lacking a cell phone, unwillingness to provide contact information, or undergoing testing at non-medical sites such as prison health facilities or antenatal care clinic.

### Data Collection Tools and Processes

Trained fieldworkers invited potential participants to enrol in the study while they waited in the HIV testing queues at the study facilities. Quantitative data were collected using electronic questionnaires built into the Research electronic data capture (REDCap)-based tablets [31] available in isiZulu and English. The questionnaires, which were either self-administered or completed with assistance from the fieldworkers, took approximately 45–60 min to complete and captured a range of information, including demographic details, socio-economic status, reasons for HIV testing, and experiences of intimate partner violence. Participants’ HIV status was unknown at enrolment for ethical reasons, and HIV test results were later obtained from clinic records.

Participants who tested positive were tracked through TIER.Net [28] and the National Health Laboratory System’s (NHLS) TrakCare system [32], also known as the central data warehouse (CDW) to monitor CD4 counts, viral load measurements, and ART initiation. The NHLS’ probabilistic linkage algorithm was used to match data using personal identifiers (IDs) such as name, date of birth, and testing facility. All data were de-identified and merged using unique enrolment IDs in Stata 17.0 for analysis [33].

For the qualitative arm, interviewers received an intensive two-week training focused on interview techniques and understanding barriers and enablers to care linkage. Using an interview guide developed by senior investigators, interviewers explored participants’ experiences with HIV testing, disclosure, treatment uptake, stigma, and available support. The guide was translated into IsiZulu, piloted, and refined for credibility. In-depth interviews (IDIs) were conducted at participants’ homes or clinics, based on their preference, with informed consent obtained. Most interviews were conducted in IsiZulu, lasted 20 to 60 min, and were audio-recorded with participants’ permission. All interviews were transcribed verbatim and translated into English [16].

Each participant was assigned a unique study identifier (e.g., KEMG_NL_F29), which was included at the end of each quote to provide context. The first part of the code refers to the facility where the participant was interviewed. The letters ‘L’ and ‘NL’ indicate whether the participant linked to care (‘L’) or did not link to care (‘NL’). This is followed by the participant’s gender (M for male, F for female) and their age at the time of the interview.

### Data Analysis

Data were exported from REDCap [31], and mortality and laboratory results were extracted from the NHLS CDW [32] using Excel. Analyses accounted for the one-stage cluster design using the svyset command in STATA 17.0 [33]. Health facilities served as the primary sampling units (clusters), stratified by facility type, and no finite population correction was applied.

Categorical variables were adjusted using the Rao–Scott second-order correction [34] and are reported as proportions with 95% confidence intervals (CIs), while skewed continuous variables are summarized as medians with interquartile ranges (IQRs). Linkage to care was expressed as a proportion of the HIV-positive cohort. Differences in continuous variables were assessed using the Mann–Whitney test, and associations between categorical variables were evaluated using Pearson’s chi-squared test [20]. Factors associated with linkage to HIV care were further examined using Pearson’s chi-square test and logistic regression models. We performed a complete case analysis, reporting the varying denominators and confidence intervals. Statistical significance was defined as p-values ≤ 0.05. The study only reported the unadjusted models, as we set out to understand the raw associations as at baseline for the total effects of the covariates/risk factors, offering an opportunity to triangulate and validate qualitative narratives, thereby strengthening findings.

For the qualitative data, we conducted a thematic content analysis [35] focused on key research questions. Two team members independently reviewed all transcripts to understand their content and scope and collaboratively developed a structured coding framework. The coded data were then linked to broader emerging themes across interviews. IDI data were manually coded by two experienced qualitative researchers using a comparative analytic approach to ensure accuracy. This analysis was guided by the interview questions, relevant literature, and an inductive approach [36].

Quantitative factors influencing linkage to care and themes from qualitative data were organized using the adapted Framework for a Systems Approach to Health Care Delivery (FSAHD) [37] across three levels: individual, family/community, and health system factors. The findings were then triangulated to provide a comprehensive understanding of facilitators of timely linkage to HIV care.

### Ethical Consideration

Ethical approval for this study was granted by the South African Medical Research Council (SAMRC) ethics committee (EC021-7/2016) and the KwaZulu-Natal provincial Department of Health. Informed consent was obtained from all participants, with separate consent required before participating in either arm of the study. Participant confidentiality was maintained by using study codes rather than personal identifiers. The decipher code sheet is securely stored, encrypted, on the principal investigator’s computer.

## Results (Quantitative Arm)

### Source Population

A total of 5,637 participants were recruited after screening 6,126 for eligibility. Of those screened, 0.9% (53/6,126) did not meet the eligibility criteria, 7.1% (436/6,126) had data quality issues, 4.3% (265/6,126) did not test, and 0.5% (31/6126) lost their test results (Fig. 2). The HIV-negative cohort constituted 74% (4,147/5,637) of the enrolled study participants, while the HIV-positive cohort constituted 21% (1,194/5,637) of the enrolled participants. The current analysis focuses on the 1,194 participants who tested HIV-positive.

### Linkage to Care

Table 2 indicates that of the 1,194 participants diagnosed with HIV at baseline, 987 (83%) were successfully linked to care within three months and tracked in the TIER.Net database, while 207 (17%) were not linked during this period. Most participants were recruited from primary health care clinics (66%; 793/1,194), followed by gateway clinics (19%; 229/1194), hospitals (9%; 105/1,194), and mobile clinics (6%; 67/1,194). The cohort was predominantly female, making up 71% (851/1,194). Most participants had at least a high school education (93%; [(665 + 434)/1,177], and over half used public transport to access health facilities (52%; 613/1,177).

**Table 2:** Socio-demographic characteristics of the HIV-positive participants at baseline and linkage in care at 3 months of follow-up in the linkage to care study in uThukela district, 2019 (N = 1194)

Variable	Total (*N* = 1,194)			Linked in care (LiC) (*N* = 987)			Not linked in care (nLiC) *N* = 207)			*p*-value
	*n*	%	95% CI	*n*	%	95% CI	*n*	%	95% CI	
*Nationality*										
South African citizen Other SADC Other African None	1,180 11 2 1	98.8 0.9 0.2 0.1	97.1–99.5 0.3–2.6 0.04–0.7 0.01–0.7	976 9 1 1	98.9 0.9 0.1 0.1	97.3–99.5 0.4–2.3 0.01–0.9 0.01–0.9	204 2 1 0	98.6 1.0 0.5 0	94.2–99.7 0.1–6.5 0.1–4.0 -	0.57
*Facility type*										
Clinics Gateway Hospital Mobile clinic	793 229 105 67	66.4 19.2 8.8 5.6	54.3–76.7 13.4–26.7 2.4–27.1 3.2–9.7	637 190 100 60	64.5 19.3 10.1 6.1	51.8–75.5 13.1–27.3 3.0–29.5 3.3–11.0	156 39 5 7	75.4 18.8 2.4 3.4	59.5–86.5 9.5–33.8 0.3–18.0 2.0–5.6	0.06
*Sex*										
Male Female	343 851	28.7 71.3	25.9–31.7 68.3–74.1	292 695	29.6 70.4	26.9–32.4 67.6–73.1	51 156	24.6 75.4	20.1–29.8 70.2–79.9	**0.03**
*Ethnicity*										
Black African Colored/Mixed ancestry Indian/Asian	1,191 2 1	99.7 0.2 0.1	99.2–99.9 0.04–0.7 0.01–0.7	984 2 1	99.7 0.2 0.1	99.0–99.9 0.1–0.8 0.01–0.9	207 0 0	100 0 0	- - -	0.70
*Education level*										
No education Primary education High school education Post matriculation	21/1,177 57/1,177 665/1,177 434/1,177	1.8 4.8 56.5 36.9	1.0–3.3 4.0–5.8 50.1–62.7 30.1–44.2	18 50 552 353	1.8 5.1 56.7 36.3	1.1–3.2 4.2–6.3 49.7–63.5 29.2–44.0	3 7 113 81	1.5 3.4 55.4 39.7	0.3–7.4 1.7–6.6 49.1–61.6 32.0–48.0	0.59
*Age*,* median (IQR)*	1,193	30	25–37	986	30	25.0–37.0	207	31	25–38	0.75
Age categories										
18–24 years 25–29 years 30–34 years 35–49 years 50 + years	269/1,193 293/1,193 251/1,193 317/1,193 63/1,193	22.5 24.6 21.0 26.6 5.3	19.3–26.2 21.7–27.7 18.7–23.6 23.0–30.5 4.2–6.7	220 243 210 259 54	22.3 24.6 21.3 26.3 5.5	18.7–26.4 21.5–28.1 18.6–24.3 22.7–30.2 4.2–7.1	49 50 41 58 9	23.7 24.2 19.8 28.0 4.3	19.6–28.3 19.1–30.1 17.0–23.0 21.2–36.0 2.4–7.6	0.76
*Marital status*										
Married (living together) Married (living separately) Cohabiting Dating Single	68/1,168 29/1,168 178/1,168 725/1,168 168/1,168	5.8 2.5 15.2 62.1 14.4	3.7–9.1 1.4–4.3 11.1–20.6 56.4–67.4 9.4–21.4	55 25 150 593 145	5.7 2.6 15.5 61.3 15.0	3.5–9.0 1.6–4.2 11.0–21.4 55.7–66.5 10.2–21.6	13 4 28 132 23	6.5 2.0 14.0 66.0 11.5	3.3–12.3 0.5–7.7 8.1–23.2 55.5–75.1 5.1–24.0	0.67
*Access to US$14 in emergencies*										
Very/somewhat difficult Fairly/very easy	714/1,171 457/1,171	61.0 39.0	52.2–69.1 30.9–47.9	595 378	61.2 38.8	51.9–69.7 30.3–48.2	119 79	60.1 39.9	51.3–68.3 31.7–48.7	0.74
*Received child support grant*	527/1,169	45.1	42.1–48.1	438	45.2	41.6–48.9	89	44.5	36.0–53.3	0.89
*Received disability grant*	53/1,163	2.8	2.2–3.7	28	2.9	2.2–3.8	5	2.5	1.4–4.5	0.64
Food security**										
Low Medium High	201/1,174 293/1,174 680/1,174	17.1 25.0 57.9	13.1–22.1 18.0–33.5 47.3–67.9	174 239 562	17.9 24.5 57.6	13.2–23.7 18.2–32.2 46.8–67.8	27 54 118	13.6 27.1 59.3	8.7–20.6 16.0–42.1 47.1–70.5	0.43
*Worked in the last year*	620/1,168	53.1	44.1–61.9	511	52.8	43.6–61.8	109	54.5	43.0–65.5	0.70
*Area of residence*										
Rural *Urban*	406 788	34.0 66.0	14.7–60.6 39.4–85.3	336 651	34.0 66.0	15.1–60.0 40.0–84.9	70 137	33.8 66.2	11.4–67.1 33.0–88.6	0.98
*Mode of transport to facility*										
On foot Public transport Private transport Other	486/1,177 613/1,177 70/1,177 8/1,177	41.3 52.1 6.0 0.7	25.2–59.5 35.3–68.4 3.9–9.1 0.2–2.1	391 523 56 6	40.1 53.6 5.7 0.6	25.3–56.9 37.3–69.1 3.8–8.5 0.1–3.0	95 90 14 2	47.3 44.8 7.0 1.0	23.9–71.9 24.7–66.7 2.4–18.5 0.2–4.2	0.48
*Time to get to the facility*										
Less than 30 min 30–60 min More than an hour	719/1,173 392/1,173 62/1,173	61.3 33.4 5.3	50.2–71.3 23.6–45.0 3.9–7.2	584 332 55	60.1 34.2 5.7	49.4–70.0 24.5–45.4 4.1–7.8	135 60 7	66.8 29.7 3.5	52.6–78.5 18.2–44.5 1.7–6.9	0.12
*Active church member*	607/1,173	51.8	45.6–57.9	496	51.1	44.3–57.9	111	54.7	45.0–57.9	0.49
*Club or society member*	212/1,175	18.0	13.8–23.3	174	17.9	13.7–23.0	38	18.8	12.8–26.8	0.71
*Currently studying*	140/1,150	12.2	9.9–14.9	117	12.3	9.8–15.4	23	11.5	8.0–16.2	0.72
*Reason for HIV testing*										
Fell ill Someone suggested it Suspected to be infected Opportunity at the facility No reason Wanted to know status Work requirement	355/1,113 85/1,113 114/1,113 75/1,113 158/1,113 323/1,11333/1,113	31.9 7.6 10.2 6.7 14.2 29.0 0.3	26.2–38.1 4.5–12.8 7.4–14.1 4.4–10.2 7.8–24.4 17.6–43.9 0.1–0.8	303 74 97 55 127 268 3	32.7 8.0 10.5 5.9 13.7 28.9 0.3	26.4–39.7 4.7–13.2 7.5–14.5 3.7–9.3 7.6–23.5 17.4–43.9 0.1–1.0	52 11 17 20 31 55 0	28.0 5.9 9.1 10.8 16.7 29.6 0	23.3–33.2 2.4–13.9 4.7–17.2 5.4–20.2 8.4–30.3 17.2–46.0 -	0.26
*First time HIV tester*	286/1,162	24.6	18.4–32.1	246	25.6	19.2–33.2	40	20.1	11.5–32.8	0.31
*Females who experienced emotional violence*	41/811	5.1	3.2–8.0	34	5.1	3.1–8.4	7	4.8	2.3–9.7	0.87
*Females who experienced physical violence*	10/810	1.2	0.5–3.1	10	1.5	0.6–3.7	0	0	-	0.26
*Male perpetrators of physical violence*	2/329	0.6	0.1–4.8	2	0.7	0.1–5.7	0	0	-	0.71
*Involved in a fight*	129/1,162	11.1	7.3–16.6	106	11.0	7.1–16.5	23	11.9	7.0–19.4	0.67
*Female age at sex debut*										
12–16 years ≥ 17 years	81/268 187/268	30.2 69.8	23.0–44.4 62.0–76.6	63 166	27.5 72.5	21.1–35.0 65.0–78.9	18 21	46.2 53.9	32.3–60.6 39.4–67.7	***0.01***
*Condom use last sex*	98/344	28.5	19.9–39.0	78	26.4	17.5–37.6	20	41.7	30.4–53.9	***0.01***
*Condom use with side partner*	102/330	30.9	21.5–42.2	81	28.5	18.8–40.8	21	45.7	34.6–57.1	***0.004***
*Frequency of condom use*										
Always Often Sometimes Never	31/343 30/343 208/343 74/343	9.0 8.8 60.6 21.6	6.2–13.0 5.1–14.5 54.1–66.8 14.8–30.3	25 28 174 68	8.5 9.5 59.0 23.1	5.9–12.0 5.6–15.6 52.4–65.2 15.4–33.0	6 2 34 6	12.5 4.2 70.8 12.5	5.5–26.0 1.4–11.5 57.4–81.4 6.4–22.9	***0.05***
*Main sexual partners in the last year*										
None 1 partner 2–10 partners	22/339 184/339 133/339	6.5 54.3 39.2	3.2–12.8 43.8–64.4 28.2–51.5	22 156 114	7.5 53.4 39.0	3.7–14.8 43.6–63.0 28.7–50.5	0 28 19	0 59.6 40.4	0 36.2–79.3 20.7–63.8	0.36
*Side sexual partners in the last year*										
None 1 partner 2–10 partners	128/340 88/340 124/340	37.7 25.9 36.5	26.5–50.4 19.6–33.4 28.3–45.5	114 79 100	38.9 27.0 34.1	27.9–51.2 21.1–33.8 26.3–43.0	14 9 24	29.8 19.2 51.1	14.4–51.7 7.6–40.4 35.2–66.8	0.11
*Sexual partners in the last year*										
None 1 partner 2–10 partners	43/336 124/336 169/336	12.8 36.9 50.3	8.9–18.2 27.4–47.6 39.4–61.2	41 109 140	14.1 37.6 48.3	9.4–20.8 28.7–47.4 38.2–58.5	2 15 29	4.3 32.6 63.0	0.9–17.7 17.6–52.3 44.7–78.3	0.10
*Males who had sex with sex worker*	22/264	8.3	5.1–13.4	19	8.4	4.9–14.2	3	7.7	2.5–21.0	0.87
*Alcohol consumption*										
Never At least once or more	770/1,158 388/1,158	66.5 33.5	62.2–70.5 29.5–37.8	641 325	66.4 33.6	62.1–70.4 29.6–37.9	129 63	67.2 32.8	59.7–73.9 26.1–40.3	0.78
*Substance abuse (drugs)*										
Never At least once or more	986/1,157 171/1,157	85.2 14.8	74.6–91.9 8.1–25.4	832 134	86.1 13.9	75.4–92.7 7.3–24.7	154 37	80.6 19.4	69.3–88.5 11.5–30.7	***0.03***

p-value of$\:\:\le\:0.05$ was considered statistically significant

p-values derived using Mann Whitney U-test for continuous data

p-values derived using Pearson’s chi-squared test and the one-stage cluster design was considered

proportions (%) for the columns reported as n/N (except if data is missing - denominator added in the n column) and the associated 95% CI

IQR – Interquartile range; CI – Confidence Interval; US$ – United States of America Dollar; LiC – Linked in Care; nLiC – Not linked in care

*z value reported

**Food security included questions on: if in the last four weeks the participant or other household members lacked food, went to sleep hungry, or borrowed. The scores ranged from 1–13, with 1–9 = low food security, 10–12 = medium food security, and 13 = high food security

Linkage to care was higher among males than females, with 85% (292/343) of the HIV-positive males linked to care compared to 82% (695/851) HIV-positive females (*p* = 0.03). Most participants (86%; 1,000/1,168) reported being in a relationship, primarily dating (62%), or cohabiting (15%). Fewer participants were single (14%), or married and living together (6%), while a small proportion of the married participants reported living separately (3%; 29/1,168). Overall, socio-demographic characteristics were similar between participants who linked to care and those who did not. However, differences were observed in facility type, sex, female age at sex debut, condom use, and drug use (Table 2).

Participants’ experience at 4-months follow-up and the proportion of patients that were linked to care at 3 months are presented in Supplement 1. Most of the participants (92%) had laboratory tests done for CD-4 count within one-month of HIV confirmation and were linked to medical care. Overall, 90% did not see any reason to join an HIV support group, possibly due to stigma or limited understanding of it benefits, while about 75% disclosed their status to supportive adult family members. Although these factors were not statistically significance, providing explicit instruction to patients about their next medical appointment at 3-months approached statistical significance (*p* = 0.09) (Supplement 1).

### Factors Influencing Linkage to Care at Three Months of HIV Diagnosis

Univariate analysis and logistic regression identified seven factors significantly associated with linkage to care at three months following HIV diagnosis: type of health facility, sex, condom use with side partner, frequency of condom use, female age at sexual debut, substance abuse (drug use), and condom use during the last sexual encounter (Table 3).

**Table 3:** Factors influencing linkage to care at 3 months after HIV positive diagnosis, uThukela district, KwaZulu-Natal, South Africa (*N* = 1,194)

Variable	*N* (%)	Unadjusted model OR (95% CI)	*p*-value
***Individual level factors***			
*Sex*			
Female	851 (71)	*1 (ref)*	
Male	343 (29)	1.29 (1.03–1.61)	***0.03***
Age (years)	1,194	1.00 (0.98–1.01)	0.75
*Age categories*			
18–24 years	269 (23)	*1 (ref)*	
25–29 years	293 (25)	1.08 (0.72–1.62)	0.68
30–34 years	251 (21)	1.14 (0.85–1.52)	0.35
35–49 years	317 (27)	0.99 (0.70–1.41)	0.97
50 + years	64 (5)	1.34 (0.60–2.96)	0.45
*Highest education attained*			
High school education	665/1,177 (57)	*1 (ref)*	
Post matriculation	434/1,177 (37)	0.89 (0.65–1.22)	0.45
Primary education	57/1,177 (5)	1.46 (0.72–2.99)	0.27
No education	21/1,177 (2)	1.23 (0.27–5.66)	0.78
*Currently studying*			
No	1,010/1,150 (88)	*1 (ref)*	
Yes	140/1,150 (12)	1.08 (0.69–1.71)	0.72
*Work in the past 12 months*			
Never	548/1,168 (47)	*1 (ref)*	
All/most months/once in a while	620/1,168 (53)	0.93 (0.65–1.35)	0.70
*Access to US$14 in emergencies* Very/somewhat difficult Fairly/very easy	714/1,171 (61) 457/1,171 (39)	*1 (ref)* 0.96 (0.72–1.27)	0.74
*Received child support*			
No	642 (55)	*1 (ref)*	
Yes	527 (45)	1.03 (0.68–1.56)	0.89
*Received a disability grant*			
No	1,130 (97)	*1 (ref)*	
Yes	33 (3)	1.16 (0.60–2.25)	0.64
*Food security* Low Medium High	201/1,174 (17) 293/1,174 (25) 680/1,174 (58)	*1 ref* 0.69 (0.34–1.38) 0.74 (0.41–1.34)	0.27 0.30
*Sexual partners in the last year*			
None	43 (4)	*1 (ref)*	
1 partner	124 (10)	0.35 (0.05–2.39)	0.27
2–10 partners	169 (14)	0.24 (0.04–1.48)	0.12
Missing	858 (72)	-	-
*Males who had sex with sex worker*			
No	242 (20)	*1 (ref)*	
Yes	22 (2)	1.11 (0.31–4.00)	0.87
Missing	930 (78)	0.79 (0.61–1.03)	0.08
*Female age at sex debut*			
12–16 years	81 (7)	*1 (ref)*	
≥ 17 years	187 (16)	2.56 (1.29–3.93)	***0.01***
Missing	926 (78)	1.29 (0.83–2.00)	0.24
*Condom use last sex*			
No	246 (21)	*1 (ref)*	
Yes	98 (8)	0.50 (0.32–0.79)	***0.01***
Missing	850 (71)	0.56 (0.40–0.78)	0.002
*Condom use with side partner*			
No	228 (19)	*1 (ref)*	
Yes	102 (9)	0.48 (0.29–0.77)	***0.01***
Missing	864 (72)	0.54 (0.40–0.72)	< 0.001
*Frequency of condom use*			
Always	31 (3)	*1 (ref)*	
Often	30 (3)	3.36 (1.63–6.93)	***0.003***
Sometimes	208 (17)	1.23 (0.54–2.80)	0.60
Never	74 (6)	2.72 (0.75–9.91)	0.12
Missing	851 (71)	1.04 (0.42–2.58)	0.92
*Main sexual partners in the last year*			
None	22 (2)	*1 (ref)*	
1 partner	184 (15)	1.28 (0.89–1.85)	0.17
2–10 partners	133 (11)	1.38 (0.79–2.42)	0.24
Missing	855 (72)	-	-
*Side sexual partners in the last year*			
None	128 (11)	1 (ref)	
1 partner	88 (7)	1.08 (0.36–3.22)	0.89
2–10 partners	124 (10)	0.51 (0.24–1.08)	0.07
Missing	854 (72)	-	-
*Alcohol consumption*			
Never	770/1,158 (66)	*1 (ref)*	
At least once or more	388/1,158 (34)	1.04 (0.78–1.38)	0.78
*Substance abuse (drugs)* Never At least once or more	986/1,157 (85) 171/1,157 (15)	*1 (ref)* 0.67 (0.47–0.96)	***0.03***
*First time HIV tester*			
No	876/1,162 (75)	1 *(ref)*	
Yes	286/1,162 (25)	1.36 (0.73–2.55)	0.31
***Community/social level factors***			
*Area of residence*			
Rural	406 (34)	*1 ref*	
Urban	788 (66)	0.99 (0.48–2.03)	0.98
*Marital status*			
Dating (living separately)	725/1,168 (62)	*1 (ref)*	
Married (living together)	68/1,168 (6)	0.94 (0.53–1.67)	0.83
Married (living separately)	29/1,168 (2)	1.39 (0.44–4.44)	0.55
Cohabiting	178/1,168 (15)	1.19 (0.62–2.30)	0.58
Single	168/1,168 (14)	1.40 (0.76–2.59)	0.26
*Active church member*			
No	566/1,173 (48)	*1 (ref)*	
Yes	607/1,173 (52)	0.87 (0.57–1.33)	0.49
*Active club member*			
No	963/1,175 (82)	*1 (ref)*	
Yes	212/1,175 (18)	0.94 (0.66–1.33)	0.71
*Females who experienced emotional violence*			
No	770 (65)	*1 (ref)*	
Yes	41 (3)	1.07 (0.46–2.50)	0.87
Missing	383 (32)	1.16 (0.83–1.62)	0.35
*Involved in a fight*			
No	1,033/1,162 (89)	*1 (ref)*	
Yes	129/1,162 (11)	0.91 (0.59–1.43)	0.67
***Health systems level factors***			
*Type of facility*			
Clinic	793 (66)	*1 (ref)*	
Gateway	229 (19)	1.19 (0.53–2.70)	0.65
Hospital	105 (9)	4.90 (1.87–12.82)	***0.003***
Mobile clinic	67 (6)	2.10 (0.97–4.57)	0.06
*Transport used to get to facilities*			
Foot	486 (41)	*1 (ref)*	
Public transport	613 (52)	1.41 (0.84–2.36)	0.17
Private transport	70 (6)	0.97 (0.33–2.90)	0.96
Other	8 (1)	0.73 (0.06–9.15)	0.79
*Time to get to the facility*			
Less than 30 min	719 (61)	*1 (ref)*	
30–60 min	392 (33)	1.28 (0.92–1.78)	0.13
More than 60 min	62 (5)	1.82 (0.80–4.10)	0.14

p-value $\:\le\:0.05$ was considered statistically significant

proportions (%) for the columns reported as n/N (except if data is missing - denominator added in the n column) and the associated 95% CI

OR odds ratio

Participants diagnosed in a hospital setting were nearly five times more likely to be linked to care compared to those diagnosed at a clinic (OR: 4.90, 95% CI: 1.87–12.82, *p* = 0.003). Participants diagnosed at mobile clinics also had higher odds of linkage to HIV care compared to those diagnosed at clinics (OR: 2.10, 95% CI: 0.97–4.57), although this association was borderline significant (*p* = 0.06). Males were more likely than females to link to HIV care within three months diagnosis (OR: 1.29, 95% CI: 1.03–1.61, *p* = 0.03). Female participants who started having sex at age 17 or older were nearly three times more likely to seek HIV care within three months of diagnosis compared to those whose sexual debut occurred between ages 12 and 16 (OR = 2.56, 95% CI: 1.29–3.93, *p* = 0.01). Individuals who used public transport to reach HIV testing sites also had higher odds of linking to HIV treatment within three months compared to those who travelled on foot (OR: 1.41, 95% CI: 0.84–2.36), even though this association was not statistically significant.

Conversely, participants who reported drug use were about 40% less likely to link to care compared to those who reported non-use of drugs (OR: 0.67, 95% CI: 0.47–0.96, *p* = 0.03). Additionally, condom use during the last sexual encounter was associated with a lower likelihood of linkage to care at three months following HIV diagnosis (OR: 0.50, 95% CI: 0.32–0.79, *p* = 0.01).

Other demographic and socioeconomic factors, such as age, marital status, educational level, employment status, area of residence, and travel time to the nearest facility, were not significantly associated with linkage to care at three months in uThukela District, KwaZulu-Natal.

### Qualitative Findings

Table 4 presents the characteristics of participants in the qualitative study recruited from the 18 facilities. All 38 participants were Black Africans. The majority (63%, *n* = 24) were recruited from clinics, while a smaller proportion (11%, *n* = 4) were from hospitals, providing a diversity of context-specific experiences. Almost all participants (92%, *n* = 35) were in a relationship, either married, cohabiting, or dating, at the time of data collection. Most were female (76%, *n* = 29), about 50% (*n* = 19) completed Grade 12, and approximately 61% (*n* = 25) reported being unemployed.

**Table 4:** Socio-demographic characteristics of the qualitative participants disaggregated by linkage status

Characteristics	Linked to care n (%); *N* = 22	Not linked to care n (%); *N* = 16	Total n (%); *N* = 38
*Facility type*			
Clinic Gateway Hospital Mobile clinic	13 3 3 3	11 4 1 0	24 7 4 3
Age in years, median (IQR)	32 (24–36)	31 (22–32)	31.5 (22–36)
*Ethnicity*			
Black African Coloured/Mixed ancestry White Indian/Asian	22 0 0 0	16 0 0 0	38 0 0 0
*Sex*			
Male Female	6 16	3 13	9 29
*Marital status*			
Married Cohabitating Dating Single	2 8 11 1	1 2 11 2	3 10 22 3
*Educational level*			
Primary school/Grade 1–7 Secondary school/Grade 8–11 Matriculated/Grade 12 Post matric	2 9 11 0	0 8 8 0	2 17 19 0
*Employment*			
Employed Unemployed	10 12	3 13	13 25
*Additional income*			
Business Child support grant Disability grant	8 10 0	5 7 2	12 17 2
*Received earnings in the past 4 weeks*			
Yes No	8 14	5 11	13 25
*Access to R200 (us$15) in case of an emergency*			
Yes No	7 15	7 9	14 24

A summary of the main themes and subthemes derived from the qualitative data is presented in Table 5, including experiences with HIV testing, disclosure, treatment initiation and adherence, positive testing experiences, and HIV-related support and care. Table 5 also illustrates these themes with direct participant quotes, categorized according to the adapted Framework for a Systems Approach to Health Care Delivery (FSAHD) [37]. Collectively, the findings highlight how individual motivations, community influences, and health system practices interact to shape linkage to HIV care in this high-prevalence district.

**Table 5:** Overview of themes and subthemes with illustrative participant quotes highlighting the range of experiences influencing linkage to HIV care across individual, family/community, as well as health systems levels

Health system	Family/community	Individual
*Reasons for HIV testing*		
**• Pregnancy, ANC routine test** “It’s because I was pregnant. I was 8 months [pregnant] because I had checked the time I started to come to the clinic and they said I should come back when I start with antenatal clinic, I went back.” **(DLHC_L_F23)** “I tested because I was pregnant. It was…. Everyone who was pregnant …. What can I say? You were forced to do an HIV test if you are pregnant.” **(ESVC_L_F31)** “I ate a Russian [sausage], I suffered from allergy, the whole body. I was forced to call an ambulance at night and go to the clinic. At the clinic they tested me and said I must pee. They said I was pregnant, but I did not believe it. After one week I went to the clinic and said I am pregnant. I then requested to test. Yes, the results were positive. I then decided to protect the baby early because I know that I am pregnant, I know that I am like this [HIV+] at least let me protect him.” **(KB2M_L_25)** **• Advise of Health care providers** “Oh, my child with regards to testing, I had sores which were like a line here. When I had these sores and there were not healing, then I decided to go to the clinic. When I arrived, nurses discussed about it and the other one said it was shingles. After they discussed, the nurse who was patient took me out. She gave me those pills, and she said “eh mother please go to that house so that you can test. I am not forcing you; it’s your right whether you want to be assisted or not. But I will request that the medication that you were going to take with you to leave it here.” **(UCSC_L_F54)**	• **Information on HIV** “Indeed, I went there. When I arrived, they told me that results are positive. There were telling me because … as a ward committee I have attended these kinds of workshop which are speaking about these things, I got the information. I was not that shocked, but I realized that “I will get help because they have found the problem”. Because some come when they are very sick, and they can’t be assisted.” **(UCSC_L_F54)** • **Infected by person they cared for** “I realised…mmh there is my brother at home who is sick and taking pills. There is an incident where he was beaten so much and bleeding, we were just hearing that if someone with this disease is bleeding, you are not supposed to touch him/her. The way he was, you were not going to wait for gloves or maybe get plastic to wear in your hands. They beat him, and he was drinking too much. I helped him and touched his blood. I did not bother about testing, the reason why I went, I had endless flue.” **(UECG_L_F43)** “It’s because I was staying with my mother who was sick, and I looked after her. My mother went to Mawose and found out that she is positive. She died in 2011… I decided to check because I was the one who was bathing her, I decided to check. I was not sick; I had no problem.” **(SDKBVC_L_F27)** • **Spouse related elements**,** Health of your Partner** “You can feel it moss; you can feel it. Also, when you see that your partner is not well… You can see that 1 and 2. I tried to put things together but could not.” **(SEUWBC_L_M40)** “His status is positive because in his room I saw papers and that made me to check…Papers indicating that he was at the clinic to check…. When I saw the papers, I did not ask, I was sad and cried. I left, the following day I came to the clinic to check, and they said I am positive…Ai I did not want to talk, my sister I do not like to talk, and I don’t like argument. Maybe we fight, and I decide to keep quiet.” (**UCSC_NL_F37)**	• **Routine check-up** “I came here to the clinic…, I just told them that I want to do all tests. They did all tests. BP, diabetes, HIV, and HB blood…” **(KEMG_NL_F29)** • **HIV related symptoms** **“**Ai, I saw that I was not feeling well, and I was losing weight. I decided to go and check my status.” **(KEMG_NL_F29)** “In 2017 November, I started losing weight, … My mother said, I must go to the clinic; I went … and got tested. They told me at the clinic that I am positive, I was shocked.” **(EOWM_L_F21)** • **Prenatal care** “After one week I went to the clinic and said I was pregnant. I then requested to test. Yes, the results were positive.” **(KB2M_L_25)** • **Feeling sick** **“**I had a sore throat, then I went to the clinic and then they said I must first test….” **(BNHC_NL_M20)** I suspected myself because I was not using a condom, I then thought I am infected because I do not usually get sick. It might happen that I have this disease, but I decided that it must not get worse and end up seriously ill. I needed to get help. **BISC_L_M47** “It’s because I was not feeling okay, and … I was suspecting that I am not well because I was always sick. I then decided to go to the clinic, and I then tested.” **(UCSC_NL_F37)** “You can feel it in your body that you are beginning to have problems that you do not understand and see that…I do not usually experience such. I am a kind of person that notices minor things.” **(SEUWBC021)**
	• **Death of a family member** “I have seen it protecting life. My brother P. the one I spoke about did not make it; he died. He did not finish two weeks after he started treatment, it kicked him out.” **(SEUWBC_L_M40)** “Yes, I had it because at home there was someone who died, my brother died because of this disease. So, he was someone who was scared to go to the clinic, this thing terrified us at home. That is why when I met with these neighbours, when they told me I took them seriously because the way my brother died was painful to all of us at home.” **(SDKBVC_L_F37)**	• **Sick partner** “My husband was sick, he was very sick and then I suspected that I also have it because he is someone who likes women. So he started getting sick as someone who have flu, he has endless flu, then I walked him here to Sgweje. He came and tested, the following month I came to test and I found out that we were both sick.” (**DIGC_L_F25)**
*Barriers to linkage to HIV care*		
**• Health systems inefficiency** “What made me not to come back? I was irritated there. After checking [testing for HIV]. they said I must go and check for TB. …when I came back, they said they did not see the results, [that] I must do it [test] again. When I need to come here, it is far; I do not have money, and now I need to go back and check again. When I came to check [for] the results, they said I should come back another day and that there were no results again. I waited for a long time, and they could not get my file. They said I should start afresh and make a new file. What makes me angry are the things that I have been doing, checking and so on, when they said I should start afresh I did not have money to go back, I did not go back. I decided that at least I should go to another clinic. When I wanted my file, they said they did not see it, because it meant they do not pay attention. It is better if I go to another clinic.” **(ECHC_NL_F34)** **• Poor counselling** “She [counsellor] said to me 'You are [HIV] positive', I said 'How come?', she said 'I do not know the things you are doing' … You know she was not patient, just to explain to me…. She said, 'My sister the results are like this; nothing will change' . I said, 'all right', … when I went outside, I was confused. I asked 'How?', she said 'Take this file and go to the nurses they will explain to you' … I took my card and went out. The way I was hurt… she was supposed to explain that it’s like this and this.” **(DIGC_NL_F36)**	• **Lack of disclosure to partner or family members** “He was very sick and then I suspected that I also have it [HIV] because he is someone who likes women. So, he started getting sick as someone who had flu, he had endless flu, …. He came and tested, and the following month I came to test, and I found out that we were both sick [HIV positive].” **(DIGC_L_F25)** “Another reason [for not linking to care] is that sometimes I see people… I know. I do not know how they do this thing of disclosing that they have HIV.” **(BNHC_NL_M20)** • **Socio‑economic factors** “It happens that I do not have money for transport.” **(UCSC_NL_F25)** “I wish to [take pills] but what can I say, I am not ok because what I am afraid of my sister, is to start while I am still unemployed. Then it happens that I have a problem with food and pills will make me sick. Yes, I fear that. Sometimes it happens that the food is not enough, and I prioritize the baby. If I start taking pills when the time is 9 to take pills, I do not have something to eat. How can I take a pill, and at the end I will be forced to quit? I do not have a problem with taking pills, that is what I fear.” **(UCSC_NL_F25)** “It is difficult to come to the clinic because transport money is the problem. Sometimes if I do not have it at all and I borrow it so that I can come to clinic… No, it’s quite a distance because we pay R14”. **(BISC_NL_M31)**	• **Denialism** “. Even if I find that I have it [HIV], it is me who needs to decide whether I will take treatment or what to do. I do not believe even now ai, a whole year has passed. Last year I thought maybe I would get sick; I am still like this… Because I also believe that I do not have it [HIV]. Ok, I tested but I did not test again.” **(EWCC_NL_F32)** “… HIV is better than diabetes, when I have accepted the situation, I will go … and check again if the results are still the same,. I plan to go when I am ready. I do not have a problem with pills, just that I have not accepted the situation.” **(DLHC_NL_F24)** • **Perceived stigma** “When you check, the other one is carrying a white card and the other one is carrying a green card “we [PLHIV] are carrying green cards, …”, do you get that. Now it is seen that those who are carrying a green card, are those who are like [HIV-positive].” **(UECG_NL_M40)** “… There is no one who knows me here. I come to the clinic to do what I need to do; it is not the same if I am around my area.” **(UCSC_NL_F37)** • **Disease progression** “Another problem I have is, I do not know whether it is because I am still fine, I do not bother myself with other things like taking medication and so on. No, I never [went]. I just think because I am still all right, I do not want to take something that I will not use…” **(UECG_NL_F37)** “…I will start taking them [ARVs] when I am sick, because if I am saying that I am taking them while I am not sick, they will make me sick and how am I going to survive?” **(UCSC_NL_M38)**
**• Confidentiality concerns** “I was going to go to LH because it’s not the same as here …. At LH, they can maintain confidentiality. Here [current clinic], they cannot keep someone’s secret. Let me just keep quiet like that and say if I am ready, I will go. In actual fact, I want to understand [new status] and accept. I do not have a problem with treatment. HIV is better than diabetes, when I have accepted the situation, I will go to X and check again if the results are still the same. I do not have a problem with that; I will start treatment.” **(DLHC_NL_F24)**	• **Fear of disclosure to Employer** “I know that I need to face that man [employer], I need to carry my card so that he can see the date. We fight about the dates… I need to explain at work, that stresses me so much. **(BISC_L_M47)** “… then I had that problem. You find that to my boss I will say I am going to the doctor, then they will say “Every month?”. **(UECG_NL_M40)**	• **Preference for alternative medication** “Yes, I once tried Gambu (Traditional herbal medicine), I once bought Gambu and drank it. No, they do advertise on radios and say “Drink Gambu, it will assist with CD4 count and so on and so on.” **(UCSC_NL_M38)** “I have tried that because I want to start treatment after I have taken traditional medication first. Do you understand me? I did it, I need to finish using these things [traditional medication] then go back to the doctor.” **(UECG_NL_M40)**
“What also makes me not to collect from this clinic, which is closer to home my child, I will be open as you have said that we must be open. In that clinic, I once told XXXX, that if workers see you enter the counsellor’s room or carrying a file because they [those collecting ARVs] sit alone, they will gossip about you which is wrong because in workshops that I am attending that [HIV positive status] is a secret”. **(UCSC_L_F54)**	• **Lack of Support Groups** “No, there are none my sister, I have never seen support groups in this area or where they are meeting where you will find that they are seated and speaking about HIV, you know. When you see them speaking about it, you see that these people who are seating here are mocking each other. You can not disclose to someone you know, it means that he will put you in trouble in the future… you can see him walking here but he has HIV. You see, you cannot say that this one has it and this does not. Because the other one is mocking do not tell me anything about someone who has HIV, the other one will say “you are saying you do not have it”. You know they cannot give you support.” (**BISC_L_M47)**	“…I do not know whether it is miracle …Maybe if I can believe it can happen, … But they say… once you attend that church, given water, prayed for, because mostly it works with prayer, your problem becomes clear. So maybe it can happen that when I got tested, I was HIV [positive]… You will never know how miracles work you see.” **(UECG_NL_F37)** • **Dislike for pills** “The reason for not taking treatment is because. I have a problem with the pills; they just stop in the throat. …, the pill is not nice. Even though it helps it will not be easy to take it for life. … but I took that decision [not to link] based on these reasons.” **(UECG_NL_F37)**
*Decision to start treatment*		
**• Positive Testing experience** “Yes, I came to Bergville to check, they said I must join the queue, I joined the queue. It was alright, they treated me well and I tested. Then they told me that I am positive, I can start pills if I like. I then said I want to start taking pills, then they took blood from me and gave me pills.” **(SDKBVC_L_F27)** “I was indeed treated well because they took care of me, and they taught me that HIV does not kill.” **(DIGC_L_F25)**	• **Knowledge/Information on the disease** “My child as I have said that there are workshops that we are attending since I am in a community committee. They gave me the information; do you see that?. They explain that “if you start early, you are helped easily and become healthy more than anyone”. I said, “I have found a jackpot if it’s like this”. So, it is adding from that information which I got outside…To me it was a mixture with that information, I said “this is the big opportunity let me accept it with both hands.” **(UCSC_L_F54)**	• **Desire to live long** “I was going to end up sick. Yes, I wanted to live for a long time.” **(UECG_L_F43)** “I had a bit of information. Where I tested, they said they wrote a letter referring me there…Yes, but I was unable to show them the letter, I wanted to test again. Yes, I tested again… Yes, just to be sure.” **(UCSC_L_M23)**
**• Advise of Health professionals** “No, I started at the clinic I was attending at Phayikeni, then I stopped and started at a Gateway [clinic]. I went to the Gateway because I discovered about my sickness; I arrived at Gateway clinic; I requested to test for everything. I found out that there were diseases, on the second time it got worse since I thought it was flu whereas it was TB, then they gave me my treatment for TB. Yes, I tested in January, two years back, the time I was taking these ones for TB.” **(ELSG_L_F52)**	“No besides, at M. I had attended so many workshops because I stayed with sick people. I even got a job at the clinic…So, I had information on HIV. It was not something that I understood…” **(SEUWBC_L_M40)**	“That is why I told that lady to give me medication while I still felt fine. So that I do not start it when it is already late. That is what I feared that I do not want to start it very late. It is whether I start it or not.” **(SEUWBC_L_M40)**
**• Follow-up by health staff** “I was going to start by taking my ARV pills, I lost my phone and they kept calling me. I did not want to take medication. Eish, I was disturbed emotionally and hurt.they kept calling and saying that I must come here. They ended up coming to my house, it was a lady and another gentleman they told me that tomorrow I must come here. They visited me in November. After testing in March …I was confused, I cannot say that I was not ready, or I was ready. I was very sad my mind was also not in a good state and I was frightened.” **(BISC_NL_M31)**	• **Experience of family member** “I started HIV treatment on that day. That lady drew my blood, and it was not that bad because I have taken a decision, I know that even if it goes up today you will never know what it will be like the following week. My sister had that problem. They stopped giving her medication because her CD4 Count was high not knowing that it was going to drop. She almost died when they gave her medication. … That is why I told that lady to give me medication while I still felt fine. So that I do not start it when it is already late. That is what I feared that I do not want to start it very late. It is whether I start it or not.” **(SEUWBC_L_M40)**	• **Previous exposure to PLHIV** “It happened before, the time when people were scared to go to the clinic for treatment. It took long time but it something that we cannot forget at home, that is why when we are sick, we rush to clinic and tell my mother and be ok.” **SDKBVC_L_F37** • **Disclosure and family support** “I told my sister; she knew our mother’s [HIV] status because I was staying with her. I told her; she did not have any problem. She said to me “Z you must take pills, do not stop taking them and if they are finished, you collect them.” **(SDKBVC_L_F27)**
**• Change in the treatment guidelines** “What gave me hope is that you do not wait to test how much your CD4 count has decreased. When you have found it [HIV], you just start medication. … ‘you will take your pills now’. They were very nice and patient.”** (UCSC_L_F54)** “I was happy …It is very good to test and get medication without starting by treating TB.” **(SDKBVC_L_F37)**	• **Support from family and friends** “My background, where I come from and where I am right now. And encouragement and support from the family, friends and the pain that was felt by my family when this one died. It was so painful” **SDKBVC_L_F37.**	“Oh, eh I told them at home so that they can support me and remind me every day if I have forgotten to take medication. As I have said if I am busy, my grandfather collects it for me. If I did not tell grandfather, if I sent him to collect this medication he was going to say, “I am going to do what, where?” **(UCSC_L_F54)**

Themes and subthemes organized using the adapted framework for a systems approach to health care delivery (FSAHD) [37]

### Integration of Quantitative and Qualitative Findings

Combining quantitative survey results (Tables 2 and 3) with qualitative findings (Table 5) provides a comprehensive understanding of the multilevel factors influencing linkage to HIV care. This mixed-methods approach highlights how individual-, family/community-, and health system-level factors interact to facilitate or hinder timely engagement in care. The qualitative findings corroborated several quantitative results while also identifying additional barriers and facilitators across these levels, thereby providing explanatory context for the associations observed in the quantitative analysis.

### Individual Level

At the individual level, quantitative results showed that personal health behaviours such as substance abuse (drugs), frequency of condom use, condom use with side partners, condom use during last sex, and early sexual debut, were significantly associated with linkage to care. Although preference for traditional/spiritual healing and testing motivations were not statistically significant in the quantitative analysis, qualitative narratives highlighted how individuals’ perceptions of risk and health experiences shaped decisions to test and engage in care.

Several participants described seeking HIV testing after recognising personal risk, particularly following unprotected sex or illness. One participant, for example, explained that not using condoms led him to suspect infection and seek testing before becoming seriously ill *(BISC_L_M47)*. Pregnancy also emerged as an important entry point for testing and linkage to care, with several women reporting that they tested during antenatal visits or after suspecting pregnancy *(KB2M_L_F25; DLHC_L_F23; ESVC_L_F31)*.

Qualitative findings further showed that some individuals delayed initiating ART due to preferences for alternative treatments. Participants reported first trying traditional herbal remedies, such as *Gambu*, which is widely advertised as improving CD4 counts *(UCSC_NL_M38)*, or completing a course of traditional medicine before returning to the clinic for ART *(UECG_NL_M40)*. Others expressed hope that prayer or faith-based healing could cure HIV *(UECG_NL_F37)*. Additional psychosocial barriers identified in the qualitative narratives, but under-represented in the survey, included fear of long-term medical use, denial of HIV status *(DLHC_NL_F24; EWCC_NL_F32)*, stigma *(UCSC_NL_F37; DLHC_NL_F24)*, dislike of pills *(UECG_NL_F37)*, and delayed recognition of symptoms. Collectively, these findings suggest that personal beliefs, perceived risk, and prior health experiences, and psychosocial factors strongly influence decisions about HIV testing and timely linkage to care, complementing the patterns observed in the quantitative data.

### Community Level

Quantitative findings showed limited participation in support groups (10%) despite high rates of disclosure to family members, with generally supportive responses (75%). Household awareness of HIV status was also associated with earlier ART initiation. Qualitative findings further elaborated on these patterns, indicating that participants with supportive family environments were more likely to link to care. Family members who understood HIV and did not stigmatise the condition encouraged disclosure and treatment initiation, highlighting the important role of family support in facilitating engagement in HIV care.

Qualitative narratives further reveal that prior exposure to HIV within the family, such as caring for an ill relative or experiencing the death of a family member due to HIV, often motivated individuals to test and initiate treatment. Participants described how witnessing a family member’s illness or death reinforced the importance of early care *(UECG_L_F43; SDKBVC_L_F27)*, supporting the quantitative finding that prior exposure to PLHIV influenced treatment decisions. At the same time, participants acknowledged the limited availability of community-based support groups as a gap in HIV-related support (*BISC_L_M47).* Support groups were viewed as valuable platforms for shared experiences, psychoeducation, and emotional support, fostering a sense of belonging and empowerment among people living with HIV.

Qualitative narratives also highlighted barriers not captured in the quantitative analysis. Stigma, secrecy around HIV status, and mistrust within intimate relationships sometimes hindered timely linkage to care *(UCSC_NL_F37; SEUWBC_L_M40)*. Participants reported feelings of shame associated with their diagnoses and concerns about the visibility of the HIV patient card, (whose colour is widely recognised in the community), which sometimes led to concealment of their HIV status and delayed treatment initiation. Internalized stigma was further reinforced by community misconceptions about HIV and fears of judgment or discrimination. Some participants expressed reluctance to disclose their status due to concerns about gossip and negative labelling within their social networks (*BISC_L_M47; UECG_NL_M40)*.

### Health System Level

Health system inefficiencies, suboptimal counselling, and concerns about confidentiality emerged as major barriers to linkage to care. Quantitative results showed that participants diagnosed in hospitals were more likely to link to care than those diagnosed at clinics, highlighting the critical role of facility type in timely linkage. Most participants (92%) completed CD4 testing within one month, while shorter travel distance to clinics and positive prior experiences with healthcare providers facilitated linkage. Qualitative findings from participants who did not link to care provided deeper insight into these structural challenges. Participants described frustrations with lost clinic files, repeated diagnostic tests, missing results, and long queues at clinics *(ECHC_NL_F34)*, all of which discouraged return visits. Poor or judgemental counselling in another clinic further eroded trust and left some participants confused or distressed at moment of diagnosis *(DIGC_NL_F36).*

Concerns about confidentiality also discouraged engagement in care. Some participants avoided nearby clinics due to fears that staff or community members might disclose their HIV status, particularly in small communities where patients and healthcare providers often know each other. Participants noted that being seen entering counselling rooms or carrying HIV-related files could lead to gossip and unintended disclosure *(DLHC_NL_F24)*,* (UCSC_L_F54)*. Conversely, respectful and supportive interactions with healthcare providers were reported as motivating factors for initiating treatment *(SDKBVC_L_F27).* Participants also emphasised that updated treatment guidelines under UTT facilitated rapid linkage *(UCSC_L_F54)*,* (SDKBVC_L_F37)*.

## Discussion

This mixed-methods study explored contextual factors influencing linkage to care following HIV diagnosis among adults in uThukela district, South Africa. The study provides a comprehensive understanding of the multilevel factors influencing linkage to HIV care by integrating quantitative and qualitative findings. Overall, linkage to care within the first three months of HIV diagnosis was 83% among study participants, higher than that reported in other African countries such as Tanzania (68%) [38], including findings from the same KwaZulu-Natal province in 2018 (71%) [39], and Cape Town in 2018 (63.1%) [40]. However, nearly one in five participants did not link within three months, highlighting persistent gaps in achieving universal access to antiretroviral therapy (ART).

At the individual level, both data strands revealed that perceived health, sexual relationships, and self-efficacy influenced linkage to care. Quantitative results showed that behavioural factors such as substance use, condom use patterns, and age at sexual debut, were significantly associated with linkage. Females who initiated sexual activity at age 17 or older were more likely to link to care, suggesting that early sexual debut may correlate with increased vulnerability, reduced autonomy, and lower health-seeking behaviour. These findings are consistent with prior studies linking early sexual initiation to structural vulnerabilities and reduced engagement with prevention and care services [41].

Qualitative narratives reinforced these patterns, revealing that individual risk perception and lived experiences strongly motivated HIV testing and linkage behaviours. Participants often sought HIV testing and care after recognising risk through unprotected sex, pregnancy, or illness. Pregnancy is a key entry point into HIV services, consistent with evidence that antenatal care remains a critical platform for HIV diagnosis and ART initiation among young women [42]. Conversely, psychosocial barriers, such as denial, asymptomatic infection, use of alternative treatments and fear of stigma, delayed care-seeking. These findings illustrate how psychosocial and cultural beliefs intersect with treatment decisions echoing prior evidence showing that psychosocial readiness and perceived vulnerability strongly influence linkage [32, 43].

Alternative treatment preference, such as traditional or spiritual healing, though not statistically significant in the quantitative analysis, emerged as a key barrier in the qualitative findings. Participants often delayed ART initiation while pursuing traditional remedies or faith-based healing, reflecting Zulu cultural practices of pluralistic health-seeking behaviour as described by Appelbaum Belisle et al. (2007) [44]. Such practices may delay timely ART initiation and disrupt continuity of care.

The integration of findings highlights the importance of HIV related knowledge, perceived health status and psychosocial readiness. While the quantitative data shows that individuals that were diagnosed from hospitals and symptomatic were more likely to initiate care, qualitative narratives illustrated how “feeling sick” enhanced linkage and ‘feeling healthy” reduced urgency and reinforced delays in seeking care. At the same time participants that had prior exposure to HIV information or who have family members that were HIV positive were more likely to link promptly to care, suggesting that knowledge and lived experience play an important/reinforcing role. The convergence of findings across methods therefore suggests that improving knowledge about HIV and ART alone is insufficient; interventions must also address emotional readiness and self-efficacy.

Substance use emerged as another significant barrier to timely linkage in the quantitative analysis. Individuals reporting drug use had lower odds of linking to care within three months of diagnosis, a finding consistent with global evidence identifying substance use as a strong predictor of delayed engagement in HIV care [45, 46]. Substance abuse has been shown to impair health-seeking behaviour, and reduce psychological readiness to initiate ART, thereby decreasing the likelihood of timely linkage to care [47, 48]. Recent reviews further confirm that substance use remains a persistent barrier to linkage, retention, and ART adherence across different settings [49, 50]. These findings highlight the need for targeted and integrated interventions that address substance use as part of HIV care strategies in high-burden settings.

Family and social dynamics also influenced linkage behaviour. Quantitatively, disclosure to family and supportive reactions were associated with improved linkage. Qualitative data contextualized these findings, showing that family support, through encouragement, reminders, and accompaniment to clinics, facilitated engagement, while fear of blame or rejection hindered disclosure, particularly among women. Participants with supportive families not only received help in remembering appointments or taking medication but also experienced a sense of belonging and validation, thus reducing internalised stigma. These findings align with literature emphasising the protective role of family involvement and social support networks in promoting linkage, adherence, and improving clinical outcomes [51, 52]. In contexts where familial misconceptions prevail, disengagement from care becomes almost inevitable. Exposure to HIV-related illness or death in family members also heightened perception of treatment benefits, echoing prior findings that social learning and emotional proximity to illness foster proactive care-seeking [53, 54].

Despite high levels of family disclosure, few participants engaged in support groups, reflecting persistent stigma and limited integration of psychosocial support within care pathways. Qualitative accounts indicate that community-level stigma, social labelling, and fear of discrimination discouraged open discussion of HIV status, reinforcing stigma as a key barrier to linkage to care and treatment continuation. These feelings align with existing evidence that stigma, disclosure challenges, and fear of community gossip continue to hinder progress across the HIV care cascade [55–57]. They highlight the importance of psychosocial and relational support in facilitating early engagement with HIV care. Strengthening community-based stigma reduction efforts, integrating peer navigation models, and couple-focused counselling may enhance social support structures and improve timely linkage to care [58].

At the health system level, facility type emerged as a strong predictor of linkage to care, with participants diagnosed in hospitals more likely to link to care than those diagnosed in clinics. Hospitals typically offer same-day ART initiation, integrated laboratory services, and more specialized staff, which may streamline the care cascade [9]. Mobile clinics also enhance access, likely due to shorter waiting times and reduced stigma associated with attending HIV-specific clinics [59–61]. Qualitative findings contextualised these results, revealing clinic-level inefficiencies, such as long waiting time, lost patient files, repeated diagnostic procedures, missing results, and provider attitudes as key barriers influencing participants’ decisions to return to certain clinics to initiate care. Similar challenges have been documented elsewhere, where administrative inefficiencies and poor service delivery hinder engagement with care [62, 63]. Participants also reported inadequate counselling on HIV testing, and treatment initiation, which undermined engagement. This supports evidence that patient–provider trust and communication are central to successful linkage [15, 64].

Concerns about confidentiality further compounded these barriers, particularly in smaller communities where fears of unintended disclosure discouraged use of nearby clinics. This aligns with findings by Bayisa et al. (2022), which showed that perceived breaches of confidentiality reduce service uptake in rural settings [65]. These gaps underscore systemic limitations in service delivery, and patient-provider interaction, underscoring the need for proactive counselling and shared decision-making to enhance linkage to and retention in HIV care.

### Implications for Policy and Practice

The findings of this study have important implications for HIV care policy and practice in high-prevalence settings such as South Africa. Firstly, facility type emerged as a key predictor, with hospitals and mobile clinics facilitating higher linkage to care. Strengthening primary health care clinics to match the accessibility, efficiency, and supportive environment of hospitals can improve timely linkage.

Secondly, strengthening patient-centred counselling and provider communication is essential for building trust and supporting early and sustained engagement in care. Training health workers in respectful, non-coercive approaches may help address negative experiences that undermine linkage.

Thirdly, the results also highlight the importance of family and community support systems in enabling disclosure, and sustained retention. Policies that integrate psychosocial support, community mobilization, and peer-led support initiatives may enhance resilience against stigma and relationship-related barriers.

Finally, addressing structural barriers such as transport costs and long waiting times requires further decentralization of services and investment in differentiated service delivery models. Expanding mobile and community-based clinics, alongside integrating HIV care with other health services, may help close the gap between diagnosis and long-term engagement in care.

### Strengths and Limitations

A key strength of this study is its mixed-methods design, which combined quantitative survey data with qualitative narratives to provide a comprehensive understanding of factors influencing linkage to HIV care. The use of both methods allowed us to capture not only measurable patterns of linkage but also the lived experiences, motivations, and challenges that explain those patterns. Furthermore, applying a systems approach to health care delivery framework allowed for a structured analysis of individual, community, and health system factors, highlighting the multi-level nature of HIV care engagement.

However, several limitations should be noted. First, the quantitative sample size was modest, and missing responses reduced the power to detect associations for some variables. The small number of male participants (29%) limited the ability to explore gender differences in depth, despite evidence of gender disparities in HIV care engagement. Second, the self-reported nature of the survey may have introduced recall or social desirability bias, particularly regarding sensitive behaviours such as disclosure and condom use. Third, the qualitative interviews were limited to those who either linked to care or did not, which may underrepresent the perspectives of those who initially linked but later disengaged from the care cascade. Finally, as the study was conducted in a single high-prevalence district, findings may not be fully generalizable to other settings in South Africa or sub-Saharan Africa with different health system configurations or sociocultural contexts.

Despite these limitations, the integration of quantitative and qualitative evidence provides a nuanced understanding of HIV linkage in the UTT era and highlights critical areas for targeted intervention.

## Conclusion

This study provides valuable insights into the complex, multilevel interplay of behavioural, psychosocial, structural, and contextual factors influencing linkage to HIV care in a high-prevalence district of South Africa. While the quantitative analysis identified key predictors of linkage, the qualitative evidence revealed the underlying mechanisms through which these factors operate.

Addressing gaps in linkage to care therefore requires a comprehensive, multilevel approach that extends beyond biomedical access to include psychosocial support, stigma reduction, and active family and community engagement. Strategies that strengthen patient-centred care, and foster trust in health services are particularly critical. Furthermore, sustained monitoring of linkage outcomes, combined with responsive and adaptive health system improvements, will be essential for achieving the UNAIDS 95-95-95 targets and ensuring that individuals not only undergo HIV testing but are also successfully linked to and retained in long-term care.

## Supplementary Information

Below is the link to the electronic supplementary material.

Supplementary material 1 (DOCX 32.2 kb)
