# Supplementary material for: Multilevel Factors Influencing Linkage to HIV Care in a High HIV Prevalence District in South Africa: A Mixed Methods Approach
Source: AIDS Behav. 2026 Apr 21;30(11):3585–605. doi: 10.1007/s10461-026-05140-3 (PMC13727956; doi:10.1007/s10461-026-05140-3)
Supplement: Supplementary file 1 [file 10461_2026_5140_MOESM1_ESM.docx]

**Supplement 1: Participant experiences during the 4-month follow-up and linkage to care at 3 months in uThukela district in 2019 (N=901)**

| *Variable* | *Total*  *(N=901)* | | *Linked to care (LiC)*  *(N=783)* | | *Not linked to care (nLiC)*  *(N=118)* | |  |
| --- | --- | --- | --- | --- | --- | --- | --- |
|  | ***n*** | ***% (95% CI)*** | ***n*** | ***% (95% CI)*** | ***n*** | ***% (95% CI)*** | ***p-value*** |
| Time to get to the facility  Less than 30 minutes  30-60 minutes  More than an hour | 531/886  307/886  48/886 | 59.9 (47.5-71.2)  34.6 (23.8-47.4)  5.4(3.6-8.1) | 451/771  275/771  45/271 | 58.5 (46.3-69.7)  35.6 (24.8-48.2)  5.8 (3.9-8.7) | 80/115  32/115  3/115 | 69.6 (54.1-81.6)  27.8 (15.7-44.4)  2.6 (0.9-7.1) | ***0.03*** |
| Where was the CD4 test done  PHC clinic  PHC CHC  PHC gateway  District or regional hospital  Mobile clinic | 465/662  56/662  90/662  8/662  43/662 | 70.2 (56.2-81.3)  8.5 (1.7-33.0)  13.6 (7.8-22.6)  1.2 (0.6-2.6)  6.5 (3.1-13.0) | 390/573  53/573  80/573  7/573  43/573 | 68.1 (53.3-79.9)  9.3 (1.9-34.5)  14.0 (7.4-24.7)  1.2 (0.5-2.8)  7.5 (3.6-14.9) | 75/89  3/89  10/89  1/89  0 | 84.3 (67.8-93.2)  3.4 (0.4-24.5)  11.2 (3.8-28.8)  1.1 (0.2-6.7)  0 | 0.26 |
| Blood taken for the CD4 count?  Within 1 month  Within 3 months  Within 12 months | 604/662  51/662  7/662 | 91.2 (84.1-95.4)  7.7 (3.8-14.8)  1.1 (0.5-2.3) | 525/573  42/573  6/573 | 91.6 (84.6-95.6)  7.3 (3.5-14.8)  1.0 (0.5-2.3) | 79/89  9/89  1/89 | 88.8 (75.5-95.3)  10.1 (4.3-21.9)  1.1 (0.1-11.2) | 0.66 |
| How difficult it is to get CD4 results  Very easy/Easy  Hard/ Very hard | 445/615  170/615 | 72.4 (65.6-78.2)  27.6 (21.8-34.4) | 378/532  154/532 | 71.1 (64.4-76.9)  28.9 (23.1-35.6) | 67/83  16/83 | 80.7 (68.0-89.2)  19.3 (10.8-32.0) | ***0.05*** |
| Partner reaction to status  Didn’t disclose  Discriminatory  No different  Supportive | 211/888  28/888  56/888  593/888 | 23.8 (19.8-28.3)  3.2 (1.7-5.7)  6.3 (3.8-10.4)  66.8 (60.3-72.7) | 186/773  25/773  51/773  511/773 | 24.1 (20.3-28.3)  3.2 (1.8-5.6)  6.6 (3.8-11.2)  66.1 (60.2-71.5) | 25/115  3/115  5/115  82/115 | 21.7 (12.6-34.9)  2.6 (0.8-8.1)  4.3 (1.8-10.2)  71.3 (56.4-82.7) | 0.62 |
| Adult family member reaction  Didn’t disclose  Discriminatory  No different  Supportive | 190/891  9/891  28/891  664/891 | 21.3 (17.6-25.6)  1.0 (0.4-2.3)  3.1 (1.8-5.5)  74.5 (69.7-78.8 | 163/776  9/776  26/776  578/776 | 21.0 (17.0-25.6)  1.2 (0.5-2.6)  3.4 (1.7-6.4)  74.5 (69.1-79.2) | 27/115  0  2/115  86/115 | 23.5 (15.3-34.3)  0  1.7 (0.4-7.2)  74.8 (64.0-83.2) | 0.55 |
| Child reaction to status  Didn’t disclose  Discriminatory  No different  Supportive | 519/890  6/890  38/890  327/890 | 58.3 (50.8-65.5)  0.7 (0.3-1.7)  4.3 (2.1-8.3)  36.7 (29.5-44.7) | 453/774  5/774  35/774  281/774 | 58.5 (50.5-66.1)  0.7 (0.2-1.8)  4.5 (2.3-8.9)  36.3 (28.5-44.9) | 66/116  1/116  3/116  46/116 | 56.9 (46.3-66.9)  0.9 (0.1-6.4)  2.6 (0.7-9.5)  39.7 (30.0-50.2) | 0.74 |
| Friends’ reaction to status  Didn’t disclose  Discriminatory  No different  Supportive | 566/889  5/889  34/889  284/889 | 63.7 (56.0-70.7)  0.6 (0.2-1.7)  3.8 (1.8-8.0)  32.0 (24.3-40.7) | 496/773  5/773  31/773  241/773 | 64.2 (57.1-70.7)  0.6 (0.2-1.9)  4.0 (1.9-8.5)  31.2 (23.9-39.6) | 70/116  0  3/116  43/116 | 60.3 (44.2-74.5)  0  2.6 (0.5-11.9)  37.1 (22.2-54.9) | 0.63 |
| Co-worker reaction to status  Didn’t disclose  Discriminatory  No different  Supportive | 730/867  1/867  23/867  113/867 | 84.2 (79.9-87.7)  0.1 (0.0-0.9)  2.7 (1.1-6.5)  13.0 (9.6-17.4) | 628/753  1/753  21/753  103/753 | 83.4 (79.8-86.5)  0.1 (0.0-1.0)  2.8 (1.2-6.4)  13.7 (10.5-17.7) | 102/114  0  2/114  10/114 | 89.5 (80.2-94.7)  0  1.8 (0.4-8.2)  8.8 (4.0-18.0) | 0.25 |
| Have you been saying it’s not real?  Not at all  Once/twice  Several times  Most of the time | 578/890  172/890  98/890  42/890 | 64.9 (53.8-74.7)  19.3 (14.2-25.8)  11.0 (7.3-16.4)  4.7 (2.7-8.2) | 510/774 145/774  84/774  35/774 | 65.9 (54.9-75.4)  18.7 (14.2-24.4)  10.9 (7.1-16.3)  4.5 (2.5-7.9) | 68/116  27/116  14/116  7/116 | 58.6 (40.2-74.9)  23.3 (10.8-43.2)  12.1 (6.0-22.9)  6.0 (2.6-13.2) | 0.61 |
| No money for transport  Disagree  Neither  Agree | 629/889  24/889  236/889 | 70.8 (58.6-80.6)  2.7 (1.4-5.2)  26.6 (17.1-38.9) | 547/773  24/773  202/773 | 70.8 (58.9-80.3)  3.1 (1.5-6.2)  26.1 (17.0-37.9) | 82/116  0  34/116 | 70.7 (50.6-85.0)  0  29.3 (15.0-49.4) | 0.47 |
| Prefer a traditional/spiritual healer  Disagree  Neither  Agree | 825/889  22/889  42/889 | 92.8 (85.5-96.6)  2.5 (1.1-5.5)  4.7 (2.0-10.6) | 717/773  19/773  37/773 | 92.8 (86.4-96.3)  2.5 (1.1-5.3)  4.8 (2.2-10.1) | 108/116  3/116  5/116 | 93.1 (73.7-98.5)  2.6 (0.5-12.1)  4.3 (0.9-17.8) | 0.93 |
| Don’t want to tell anyone about status  Disagree  Neither  Agree | 729/889  29/889  131/889 | 82.0 (69.7-90.0)  3.3 (1.4-7.4)  14.7 (8.1-25.4) | 627/773  26/773  120/773 | 81.1 (68.9-89.3)  3.4 (1.4-7.7)  15.5 (8.6-26.3) | 102/116  3/116  11/116 | 87.9 (64.5-96.7)  2.6 (1.3-18.2)  9.5 (3.0-26.2) | 0.42 |
| Perceptions of care | | | | | | | |
| Return for treatment education class  Yes  No | 250/661  411/661 | 37.8 (25.7-51.8)  62.2 (48.3-74.4) | 210/572  362/572 | 36.7 (26.2-48.6)  63.3 (51.4-73.8) | 40/89  49/89 | 44.9 (18.9-74.1)  55.1 (25.9-81.1) | 0.47 |
| Told to return for CD4 in 3 months  Yes  No | 445/658  213/658 | 67.6 (49.4-81.7)  32.4 (18.3-50.6) | 371/569  198/569 | 65.2 (47.7-79.4)  34.8 (20.6-52.3) | 74/89  15/89 | 83.2 (52.6-95.6)  16.9 (4.4-47.4) | 0.09 |
| Have you joined a HIV support group?  Yes  No | 89/884  795/884 | 10.1 (4.8-19.8)  89.9 (80.2-95.2) | 75/771  696/884 | 9.7 (4.9-18.5)  90.3 (81.5-95.1) | 14/113  99/113 | 12.4 (4.3-30.9)  87.6 (69.1-95.7) | 0.39 |
| Hospitalised overnight in the past year  Yes  No | 91/886  795/886 | 10.3 (8.8-11.9)  89.7 (88.1-91.2) | 74/772  698/772 | 9.6 (8.1-11.4)  90.4 (88.6-91.9) | 17/114  97/114 | 14.9 (10.7-20.4)  85.1 (79.6-89.3) | ***0.03*** |
| Criticized you  Disagree  Neither  Agree | 859/888  1/888  28/888 | 96.7 (94.4-98.1)  0.1 (0.0-0.9)  3.2 (1.8-5.4) | 746/773  1/773  26/773 | 96.5 (93.9-98.0)  0.1 (0.0-1.0)  3.4 (1.9-5.9) | 113/115  0  2/115 | 98.3 (89.1-99.7)  0  1.7 (0.3-10.9) | 0.64 |
| Guided you on what to do next  Disagree  Neither  Agree | 72/885  13/885  800/885 | 8.1 (6.1-10.8)  1.5 (0.6-3.6)  90.4 (86.4-93.3) | 67/770  11/770  692/770 | 8.7 (6.4-11.8)  1.4 (0.6-3.2)  89.9 (85.7-92.9) | 5/115  2/115  108/115 | 4.4 (1.8-10.4)  1.7 (0.2-11.1)  93.9 (85.5-97.6) | 0.25 |
| Did not understand your situation  Disagree  Neither  Agree | 701/886  16/886  169/886 | 79.1 (68.1-87.1)  1.8 (1.0-3.2)  19.1 (11.0-30.9) | 602/771  13/771  156/771 | 78.1 (66.6-86.4)  1.7 (0.9-3.0)  20.2 (11.8-32.4) | 99/115  3/115  13/115 | 86.1 (73.7-93.2)  2.6 (0.7-9.0)  11.3 (4.3-26.7) | 0.12 |
| Gave helpful information  Disagree  Neither  Agree | 69/886  13/886  804/886 | 7.8 (5.5-11.0)  1.5 (0.6-3.3)  90.7 (86.4-93.8) | 64/771  10/771  607/771 | 8.3 (5.7-12.0)  1.3 (0.5-3.0)  90.4 (85.8-93.6) | 5/115  3/115  107/115 | 4.4 (1.6-11.3)  2.6 (0.7-9.1)  93.0 (85.4-96.8) | 0.21 |
| Patient with me  Disagree  Neither  Agree | 63/886  14/886  809/886 | 7.1 (5.2-9.7)  1.6 (0.7-3.6)  91.3 (87.6) | 58/771  11/771  702/771 | 7.5 (5.4-10.4)  1.4 (0.6-3.4)  91.1 (87.0-93.9) | 5/115  3/115  107/115 | 4.4 (2.0-9.1)  2.6 (1.0-6.8)  93.0 (88.9-95.7) | 0.12 |

p-value of$\leq0.05$ was considered statistically significant

p-values derived using Pearson’s chi-squared test and the one stage cluster design was considered

proportions (%) for the columns reported as n/N (except if data is missing - denominator added in the n column) and the associated 95% CI
